# Core-shell-structured silica/polyacrylate particles prepared by Pickering emulsion: influence of the nucleation model on particle interfacial organization and emulsion stability

**DOI:** 10.1186/1556-276X-9-534

**Published:** 2014-09-29

**Authors:** Jing Ji, Shi Shu, Feng Wang, Zhilin Li, Jingjun Liu, Ye Song, Yi Jia

**Affiliations:** 1 State Key Laboratory of Chemical Resource Engineering, Beijing University of Chemical Technology, Beijing 100029, China; 2 Beijing Key Laboratory of Electrochemical Process and Technology for Materials, Beijing University of Chemical Technology, Beijing 100029, China

**Keywords:** Polymer composites, Wetting ability, Nucleation model, Core-shell, Pickering emulsion, Self-assembly

## Abstract

This work reports a new evidence of the versatility of silica sol as a stabilizer for Pickering emulsions. The organization of silica particles at the oil-water interface is a function of the nucleation model. The present results show that nucleation model, together with monomer hydrophobicity, can be used as a trigger to modify the packing density of silica particles at the oil-water interface: Less hydrophobic methylmethacrylate, more wettable with silica particles, favors the formation of core-shell-structured composite when the composite particles are prepared by miniemulsion polymerization in which monomers are fed in batch (droplet nucleation). By contrast, hydrophobic butylacrylate promotes the encapsulating efficiency of silica when monomers are fed dropwise (homogeneous nucleation). The morphologies of polyacrylate-nano-SiO_2_ composites prepared from different feed ratio of methylmethacrylate/butylacrylate (with different hydrophobicity) and by different feed processes are characterized by transmission electron microscopy (TEM) and scanning electron microscopy (SEM) techniques. The results from SEM and TEM show that the morphologies of the as-prepared polyacrylate/nano-SiO_2_ composite can be a core-shell structure or a bare acrylic sphere. The stability of resulting emulsions composed of these composite particles is strongly dependent on the surface coverage of silica particles. The emulsion stability is improved by densely silica-packed composite particles.

## Background

Since the emulsion stabilized by only fine solid particles, as described in 1907 [1], these so-called Pickering emulsions have been obtained with a wide variety of organic or mineral powders [2-12]. Very strong anchoring energy and specific interparticle interactions at interfaces are at the origin of peculiar properties of solid stabilized emulsions. Some general rules concerning solid-stabilized emulsions arise from different studies reported in the literatures [13,14]. Energy of attachment of a particle to a liquid-liquid interface is related to the interfacial tension *Υ*_OW_ and the particle contact angle *θ* at the interface. The energy needed to remove a particle of radius *b* from this interface is estimated using *E* = *πb*^2^*Υ*_OW_ (1 ± cos(*θ*))^2^, where *b* is the particle radius, *γ* is the interfacial tension, and *ϑ* is the contact angle measured across the aqueous phase [15]. The minus sign is for removal into the water phase and the plus sign is for removal into the oil phase. The maximum stability of particles at the oil-water interface is attained at 90°. To adsorb at an interface, particles need to be partly wetted by both phases. The relative balance between hydrophilic and lipophilic properties of these particles is considered to be the most important parameter governing the creation and stabilization of such emulsions [13,14].

Obviously, the affinity between inorganic (silica particle) and organic (polymers) components plays an important role in combining silica and polymeric particles, and it can be improved by modifying the surface of silica particles with coupling agents or by utilizing electrostatic attractions and/or an acid-base interaction [16-22]. To avoid the supplementary modifying stage made for silica particles, some previous experiments used unmodified solid particles to stabilize the emulsion under carefully controlled conditions. Arditty et al. [23] indicated that the surface coverage of particles increased with the increasing of the mixing intensity. Furthermore, they used different oils, with the viscosities ranging from 10 to 350 cP, to prepare the solid-stabilized emulsions, but found no apparent effect of o/w ratio on the generation of the emulsions. Hey [24] further proposed that the stability of o/w Pickering emulsion using highly viscous oils was closely related to the processing time. Since the viscous force within the oil droplets can create resistance to particle penetration into the interface, the viscosity of dispersed phase acts as a damping factor for a particle anchoring at the o/w interface and plays an important role during the emulsification process [25]. M. J. Percy and S. P. Armes [26-28] employed three different ultrafine alcoholic silica sols, with neither surface pretreatments nor auxiliary comonomers required, to successfully prepare colloidally stable inorganic/organic hybrid particles. R.F.A. Teixeira [29] discussed the hybrid ‘soft’ polymer latexes armored with Laponite XLS clay discs. The experiment showed that although the use of monomers that had high water solubility and was prone to hydrolyze under basic conditions would result in complete microcoagulation, the use of small amounts of methacrylic acid as auxiliary monomer promoted clay adhesion to the surface of the particles in the Pickering emulsion (co)polymerization of hydrophobic monomers.

Actually, if the preparing conditions (such as the viscosity of dispersed phase [24], the relative balance between hydrophilic and lipophilic properties of these particles [30-32], the polar compatibility [27] between dispersed and dispersing phases, the pH values of aqueous solutions [31], etc.) are right, untreated inorganic particles can be thermodynamically adsorbed to and anchored in the surface of the polymer particles, thus forming the core-shell-structured composite particles.

Inspired by the works of Hey [24], we investigate the effects of the glass-transition temperature Tg (i.e., viscosity of the growing particles) of growing polymer particles and the monomer mixture hydrophobicity on the anchorage of silica particles in the polymer by varying the mass ratio of MMA/BA. It is found that the effect of wetting ability of acrylic monomers on silica adsorption is not always consistent with the model [15] proposed by Hey and S. Levine for solid-stabilized emulsions. The anchorage of silica particles in the polymer also depends on the feeding processes of monomers. In the Pickering emulsion polymerization, the feeding process, dropwise or in batch, determines the types of initially prepared emulsion (i.e., the emulsion of a colloidal suspension of latex particles or of a colloidal suspension of monomer droplets), which thereby forms a conventional emulsion or a miniemulsion, respectively. The droplets in a miniemulsion are typically in the range of 100 ~ 500 nm in diameter, which are depended on the intensity of ultrasonication. The small droplet size and the consequent large droplet surface area result in most of the silica particles being adsorbed to the monomer droplets. As no surfactant is available to form micelles and stabilize aqueous phase particle nucleation, there is little or no micellar or homogeneous nucleation [33,34]. In contrast to the homogeneous nucleation of the conventional emulsion, the monomer droplets become the primary locus of particle nucleation, and droplet nucleation is predominant in miniemulsion. Different nucleation models may lead to different behaviors in viscosity of the growing particles and in hydrophobicity of monomers during the polymerization processes. Consequently, the characteristics of the final composite particles may be affected by the nucleation models. In this paper, we not only discuss the effects of feed mass ratios of MMA/BA (with varied monomer hydrophobicity) on the formation of the core-shell structure of the resulting composite particles, but also explain why the effect of wetting ability of acrylic monomers on silica adsorption is not always consistent with the model proposed by Hey and S. Levine from the nucleation mechanism perspective. Finally, we discuss how the morphologies of the composite particles influence the stability of emulsions. Understanding this mechanism is fundamental in designing composite emulsions with desired properties.

## Methods

### Materials

Silica sol, obtained from Beijing Xingdaxin Corporation, is 10 ~ 20-nm silica nanoparticles dispersed in an aqueous solution. The silica concentration is 30% by weight. Anionic initiator potassium peroxydisulfate (KPS) and acrylate monomer: methyl merthacrylate (MMA), butyl acrylate (BA) were purchased from Sinopharm Chemical Reagent Co., Ltd. All materials were used as received.

### Synthesis of polyacrylate-silica composite particles

Polyacrylate-silica composite emulsion was prepared by two different methods, which were adopted as follows: 1) the mixture of water and aqueous silica sol were agitated mechanically for 20 min, and subsequently, the solution was degassed with nitrogen gas and kept in nitrogen atmosphere. When the temperature was raised to 78°C, monomers were added dropwise into the system, and the initiator solution was added to initiate the polymerization simultaneously. 2) The nano-SiO_2_ sol mixed with acrylic monomers was ultrasonically dispersed into water for 60 min in the ice bath to produce a stable Pickering or miniemulsion, in which there was a colloidal suspension of monomer droplets stabilized by silica particles. The resultant Pickering emulsion was poured into a 100-ml three-neck flask equipped with a nitrogen inlet and a reflux condenser. The emulsion was agitated mildly and polymerized at 78°C for 8 h. The dropwise and in-bulk feeding ways of monomers were also designated as method 1 and method 2, respectively. Before characterization, samples were diluted with water for transmission electron microscopy (TEM) and scanning electron microscopy (SEM). The emulsion sample was centrifuged at 12,000 rpm for 5 min, and the sediment was isolated from supernatant for thermogravimetric (TG) measurement.

### Characterizations and contact angle measurements

A FE-JSM-6701 F SEM (JEOL, Akishima-shi, Japan) and TEM (Hitachi H-800, Hitachi, Tokyo, Japan) were used to observe the morphologies of polyacrylate/silica composite particles. The average particle diameter and the particle size distribution were measured using the dynamic light scattering technique (DLS) (Nano ZS90, Malvern, Worcestershire, UK). Before TG measurement, the samples were placed in vacuum for 24 h at 60°C and then purged by nitrogen gas.

Mixtures of MMA and BA, their mass ratios ranging from 3/1 to 1/3, were used for contact angle measurements. Liquid monomer droplets were dripped carefully onto the sample surface which was covered by a membrane derived from silica sol, and the average value of five measurements, made at different positions of the same sample, was adopted as the average values of contact angles of the substrates. The error of the mean contact angle values, calculated as the standard deviation, did not exceed 2° and 3°. Tilt angle was measured using a contact angle goniometer (JC-2000C2 China).

## Results and discussion

Since the surface of unmodified silica is hydrophilic, it is expected to stabilize the oil/water emulsion polymerization. According to the following relationship: *E* = *πb*^2^*Υ*_OW_ (1 ± cos (*θ*))^2^, 90° is the angle corresponding to the maximum energy value necessary to move the particle in which it is preferably wetted [15]. As MMA is more hydrophilic than BA, the acrylic oligomers prepared should become more compatible with hydrophilic silica with the increase of mass ratio of MMA/BA. Thus, more silica particles are expected to be incorporated in the composite particles according to the above model [15].

However, the compatibility of hydrophilic silica with acrylic monomers varies with the types of initially prepared emulsion (i.e., an emulsion of a colloidal suspension of latex particles or of a colloidal suspension of monomer droplets) which result from different feed processes. It is expected that the packing density of the silica particles at the oil-water interface can be controlled by the feeding process.

**Figure 1:**
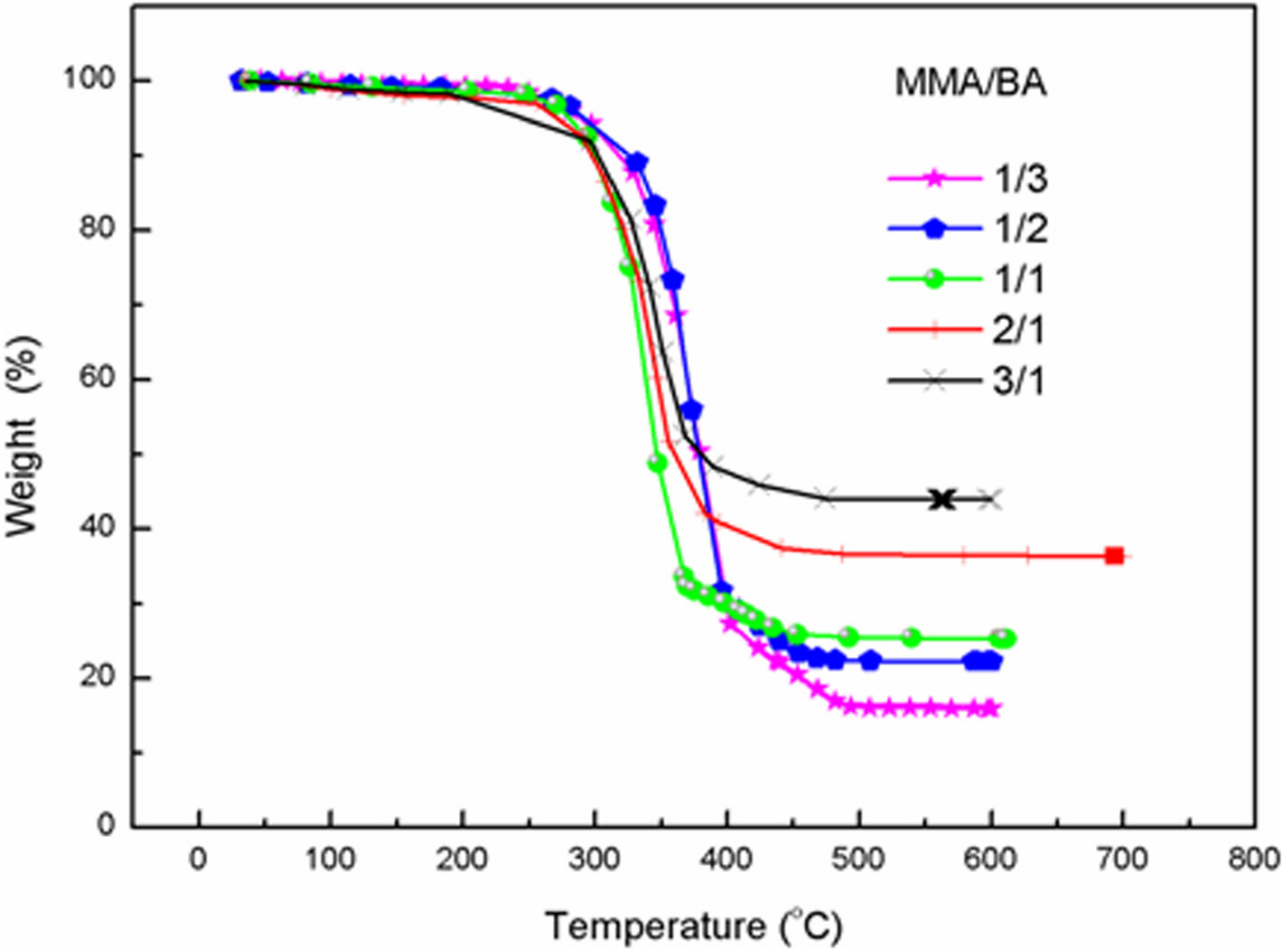
TG curves of composite emulsion with different mass ratio of MMA/BA.

**Figure 2:**
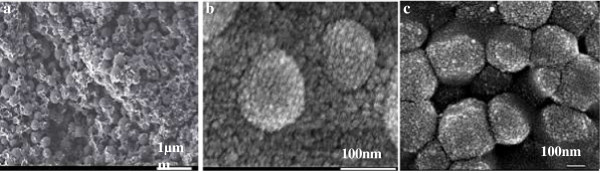
**SEM images of composite particles of different ratios of MMA/BA. (a)** 1/2, bare acrylic polymer; **(b)** 1/1, less densely encapsulated; **(c)** 2/1, well encapsulated. The composite particles were prepared by method 2.

In order to verify this hypothesis, we prepared the composite emulsion of different mass ratios of MMA/BA, and the mixtures of MMA and BA were added dropwise (method 1) or in bulk (method 2). As expected, when monomers are added in batch, the solid-stabilized emulsion prepared is a colloidal suspension of monomer droplets stabilized by silica particles. In this case, the affinity of monomer droplets with silica particles plays an important role in the formation of the core-shell structure, as indicated by Hey and S. Levine [15,17]. When the mixture of monomers added contains a higher proportion of MMA, the monomer droplets or the polymer as prepared show a greater affinity with the hydrophilic silica particles due to the hydrophilic characteristics of MMA. The influence of the ratio of BA to MMA on the affinity of polymer with silica was investigated by measuring the contact angles (see Table 1), and was confirmed by the silica content incorporated in the composite emulsion quantitatively determined by TG (as shown in Figure 1). It was assumed that major weight loss during heating was associated with thermooxidative degradation of polyacrylate, and the residue close to 700°C was sole silica. The silica content significantly increased from 15.95% to 43.85% with the increasing of mass ratio of MMA/BA from 1/3 to 3/1, which revealed that the composite particles obtained with more MMA was more compatible with inorganic silica (see Table 2).The structure of silica particles at the oil-water interface was visualized by means of SEM. Obviously, a high proportion of MMA in the monomer mixture favors the formation of core-shell structure (Figure 2).

**Table 1:** The summary of different acrylate/silica nanocomposite synthesis

**MMA/BA**	**Feed of SiO**_**2 **_**(wt.%)**	**Solid content (%)**	**Feed process**^**a,b**^	**Stability of emulsion**	**Contact angle (*θ*)**^**c**^	**Particle size**^**d **^**(nm)**
3/1	12	39	Dropwise	Yes	40°	289
2/1	12	39	Dropwise	Yes	44°	295
1/1	12	39	Dropwise	Yes	52°	267
1/2	12	39	Dropwise	Yes	60°	266
1/3	12	39	Dropwise	Yes	65°	287
3/1	8	16	In bulk	Yes		258
2/1	8	16	In bulk	Yes		262
1/1	8	16	In bulk	Yes		244
1/2	8	16	In bulk	Yes		257

^a^The emulsion was prepared by method 1 to form a colloidal suspension of latex particles; ^b^The emulsion was prepared by method 2 to form a colloidal suspension of monomer droplets; ^c^Measured at the interface between the mixture of monomers and silica sol coated surface; ^d^As determined by DLS.

**Table 2:** Effect of the MMA/BA on the polymer’s affinity with the silica

MMA/BA (mass ratio)	1/3	1/2	1/1	2/1	3/1
Content of silica (%)	15.9	21.7	27.1	36.3	43.8

**Figure 3:**
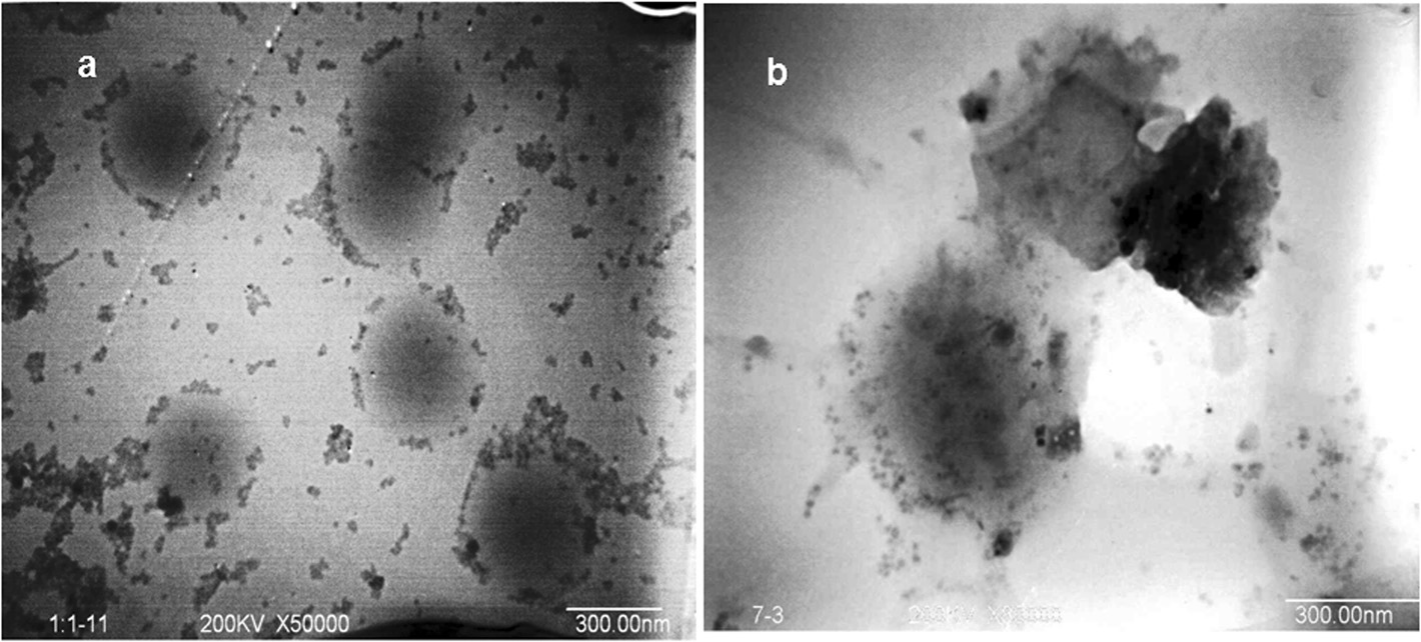
**TEM images of composite particles.** With MMA/BA of **(a)** 1/1, loosely encapsulated and **(b)** 1/2, densely encapsulated. The composite particles were prepared by method 1.

**Figure 4:**
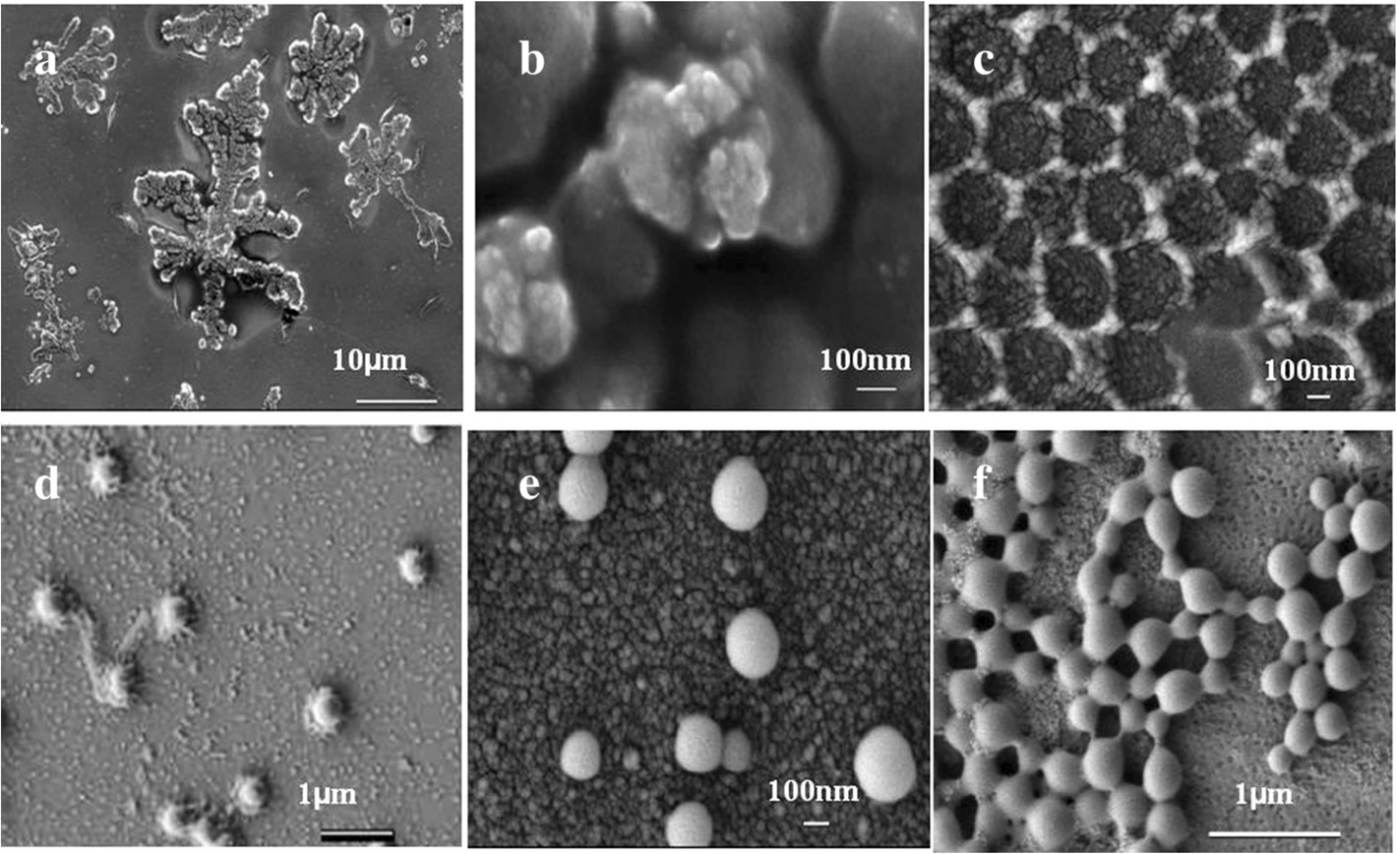
**SEM images of composite particles with MMA/BA of (a) 1/3, non-continuous film formed due to the combination of the soft acrylic polymer with the rigid SiO2, (b) 1/3, SiO2 particles incorporated in the polymer viewed at larger magnitude, (c) 1/2, well encapsulated, (d) 1/1, loosely encapsulated, (e) 2/1, bare acrylic particles, (f) 3/1, bare acrylic particles.** The composite particles were prepared by method 1.

**Figure 5:**
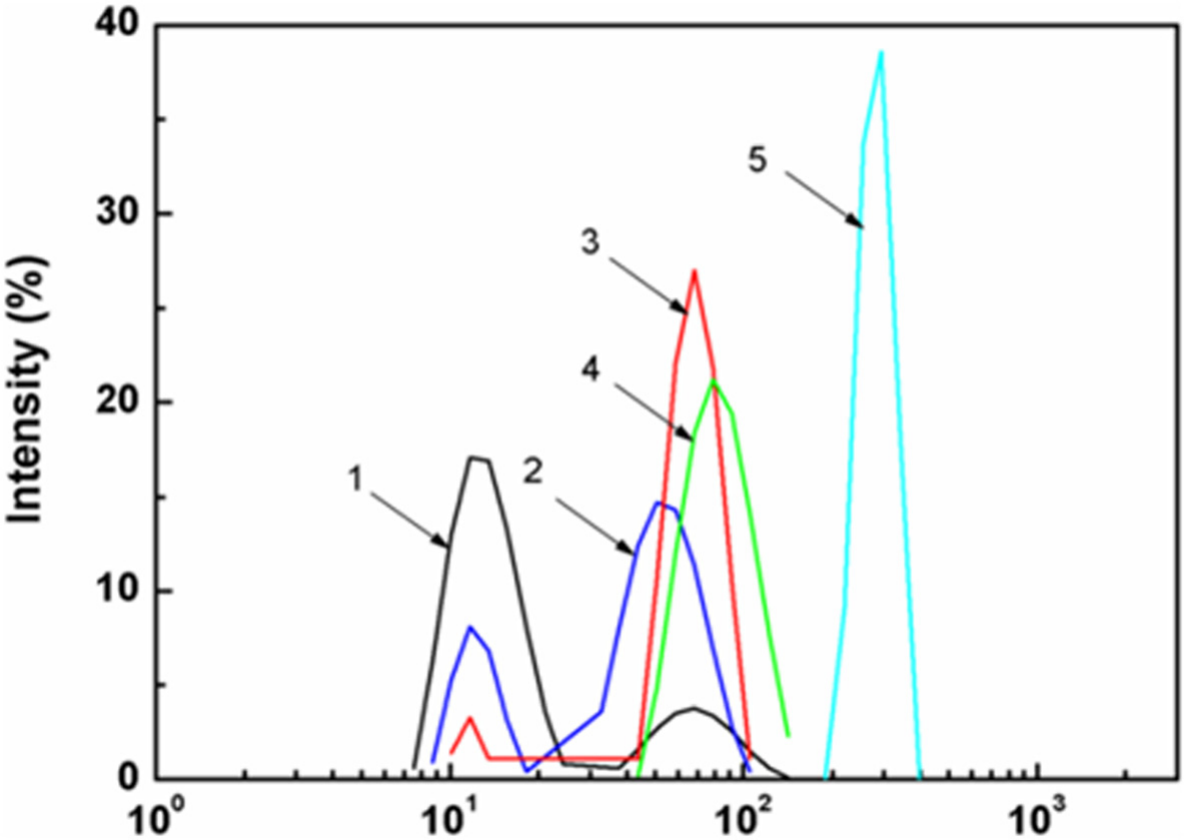
**Size distributions of polymer/silica (5 wt.%) composite particles at different polymerization times.** (1) 2 min, (2) 5 min, (3) 8 min, (4) 11 min, and (5) 311 min. Mass ratio of MMA to BA is 1/2.

Conversely, when composite emulsions were prepared by feeding monomers dropwise, the observations on the morphologies of composite particles obtained by SEM and TEM indicated that the as-prepared acrylic/nano-SiO_2_ composite particles changed from the core-shell structure to bare polyacrylate spheres with the increase of feed ratio of MMA/BA (Figures 3 and 4). Only bare polyacrylate particles were obtained when the mass ratio of MMA/BA was increased up to 2/1, which seemed to be contradictory with the rule proposed by Hey and S. Levine [15,17] (Figure 4e,g). In addition, the ratio of MMA/BA does not show significant influence on the particle size and size distribution. Previous study showed that the particle size and size distribution would rather be influenced by the amount of SiO_2_ than the ratio of MMA/BA [35].This phenomenon can be explained by the formation process of hybrid particles derived from different nucleation mechanisms. In the case of feeding dropwise, the particles are formed as in the case of conventional emulsion polymerization. In this process, the monomer is dropwise added in an aqueous solution of silica sol, and the polymerization is started by means of a water-soluble initiator system. In principle, polymer particles are formed by precipitation of growing oligomers in the aqueous phase (homogeneous nucleation) rather than entry of radicals into monomer droplets due to the relatively low probability for a radical to enter into the large (1 to 10 μm) monomer droplets. At the initial stage of polymerization, primary particles of polyacrylate forming in aqueous solution tend to absorb silica particles present to lower their surface energy. The combining process of polymer and silica is monitored by laser light scattering (see Figure 5).The first peak of bimodal curve is mainly assigned to silica particles, and the second peak represents the size of aggregation of primary polymeric particles or growing oligomers forming at the initial stage of polymerization. As shown in Figure 5 (curves 1 to 3), the intensity of the first peak lessens and disappears with the increase of reaction time, whereas the second peak intensifies. This phenomenon implies that most of the free silica particles are depleted and adsorbed to the surface of polyacrylic particles.

As indicated above, the adsorption of silica particles is at the polymer-water interface, and the composite emulsion is a colloidal suspension of latex particles stabilized by silica particles. Therefore, besides the silica's affinity with polyacrylate segment, the intrinsic viscoelasticity of the latex particles is another influencing factor on the anchorage of silica particles.

The polymer particles prepared with different mass ratios of MMA/BA have varied glass transition temperature (Tg). As the mass ratio of MMA/BA is increased, the Tg of the dispersed phase (aggregation of primary particles) increases due to more MMA incorporated therein, and the as-prepared polymer particle behaves like a rigid solid. This leads to believe that the viscosity of polymer particles or viscous forces within the growing particles can create resistance to silica particle penetration into the growing polymeric particles. When the mass ratio of MMA/BA is increased to 2/1, the viscosity of polymer particles, acting as a damping factor to silica particle penetration, is up to a point where it will eventually become a barrier to silica particle penetration. Furthermore, the active precursors or primary particles of MMA/BA of 2/1 are more hydrophilic and more compatible with aqueous system, and they can be stabilized by themselves rather than by silica particles. Therefore, it is not necessary for polyacrylic particles to absorb silica particles for their stability. In this case, the adsorption is ruled by the intrinsic viscous forces within the growing particles rather than the hydrophobicity of the polymeric segments.

**Figure 6:**
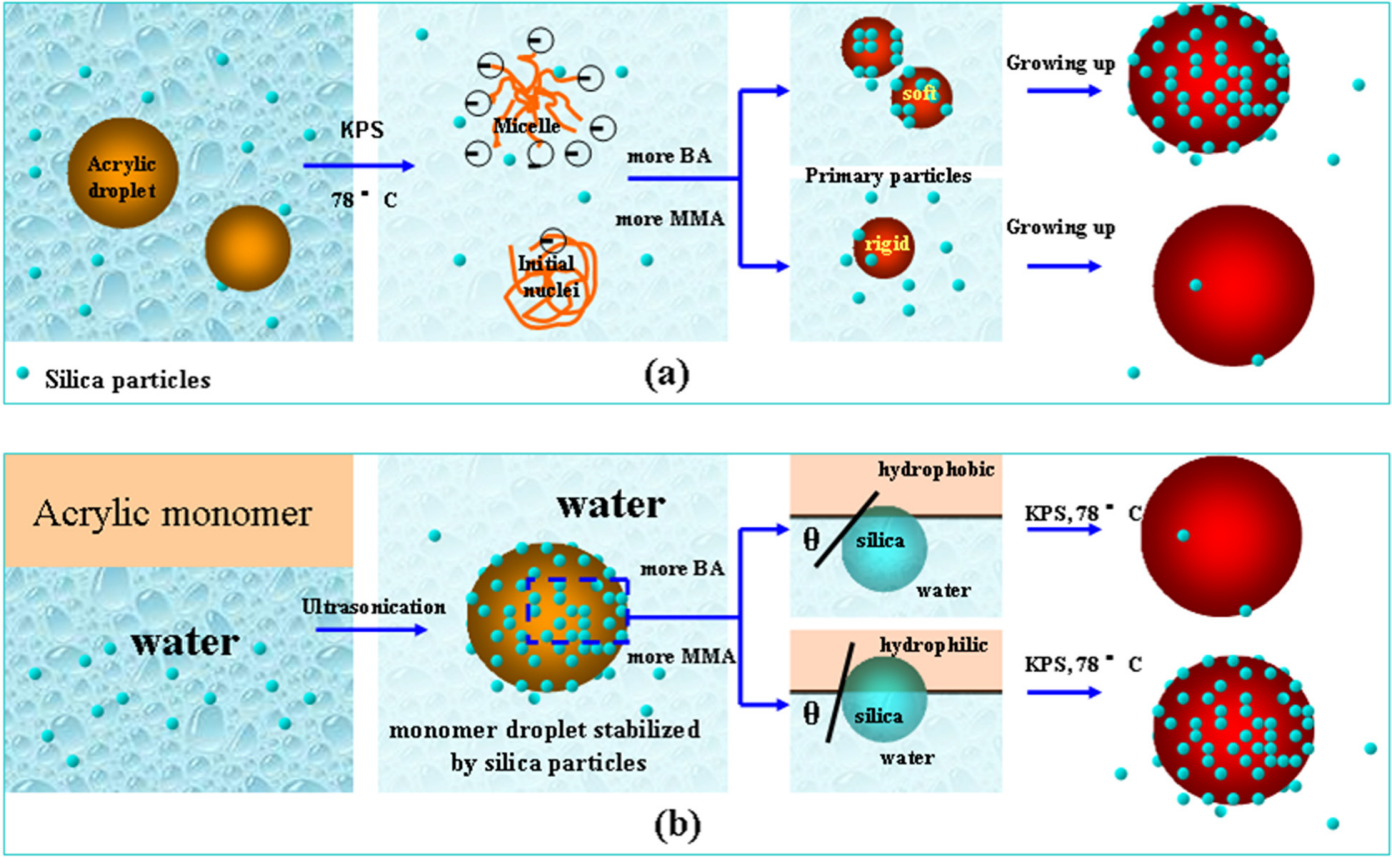
**The schematic diagram of the formation of the composite particles.** Via the means of **(a)** monomers added dropwise (homogenous nucleation) **(b)** monomers added in bulk droplet nucleation).

Whereas in the case of monomer added in bulk, a miniemulsion is prepared by high-power ultrasound of the mixture. The monomer droplets stabilized by silica particles are then obtained, which have a size between 100 and 200 nm. The formation mechanism of the composite particles formed via miniemulsion however is mainly droplet nucleation [33,34]. Droplet nucleation suggests that the droplets formed during the miniemulsification step are polymerized via radicals that enter the monomer droplets due to the statistics of radical entry and overall size, and every single droplet is nucleated. In this case, initially, the stable monomer droplets exist in the liquid state and experience lower viscous force. Thus, the hydrophilicity of monomer droplets, rather than viscosity, is predominant. The monomer droplets of a higher proportion of MMA are more compatible with silica particles, and silica particles tend to anchor into the surface of monomer droplets. Therefore, the formation of the core shell structure depends on the contact angle or affinity of silica rather than viscosity of the organic phase. The formation process of the composite particles is schematically demonstrated by Figure 6.

As the stability of Pickering emulsion derives from the solid particle absorption at the surface of the organic phase, the structure of core (polymer)-shell (silica particle) composite particles plays an important role in stabilizing the as-prepared composite emulsion. In order to investigate the effect of core-shell structure on the stability of the emulsion, composite emulsions containing different morphologies of composite particles are prepared by varying the ratio of MMA/BA in the presence of silica nanoparticles. The stability of the resultant composite emulsion is further compared with that of both the blend of polyacrylate emulsion and silica sol and the emulsion without silica. By inspection of Table 3, it is clear that the composite emulsion containing more core-shell-structured particles, especially polymerized with MMA/BA of 1/2, possesses superior store stability and Ca^2+^ stability, as compared with the others. The negatively charged initiator KPS provides a stabilizing effect on the system in emulsion polymerization, i.e., silica free run, but it is not enough to keep the long-period stability of the emulsion, which is impractical for latex applications.

**Table 3:** **Stability of polyacrylate latex, composite latex, and polyacrylate/12% SiO**_**2 **_**blend latex**

**Emulsion**	**MMA/BA (mass ratio)**	**Feed SiO**_**2 **_**(wt.%)**	**Store stability**^**c **^**(days)**	**Ca**^**2+**^**stability**^**d **^**(ml)**	**Morphology**
Composite emulsion^a^	1/3	12	50		Non-continuous film
	1/1	12	180		Loosely packed
	2/1	12	60		Bare ball
	3/1	12	30		Bare ball
	1/0	12	Aggregation		
		12	200	38	Raspberry-like
	1/2	0	60	20	
		3	150	26.2	Raspberry-like
		5	350	25.8	Raspberry-like
Blend of acrylic emulsion^b^ and silica sol	1/2	12	1	9	

^a^Solid content of emulsion is 39%, and the monomers were fed dropwise; ^b^the acrylic emulsion prepared with the MMA/BA of 1/2; ^c^The shelf life at room temperature until gelation, aggregation or delamination was observed; ^d^this is determined by the following: To 16 ml of emulsion sample was added a 0.5 wt. % of calcium chloride solution dropwise until the flocculation was observed. The least volume of the calcium chloride solution required for emulsion flocculating was designated to the Ca^2+^stability.

## Conclusions

In summary, core-polyacrylate/shell-silica nanocomposite particles are synthesized by simple soap-free emulsion polymerization employing negatively charged silica sol. It is found that the formation of polyacrylate (core)-silica (shell)-structured composite particles is obviously influenced by either the wettability of acrylic monomers or the viscosity of the dispersed phase (i.e., polymeric particles in growth), and their impacts on the morphologies of composite particles depend on the nucleation models of polymer which is also determined by feeding processes of monomers. Less hydrophobic MMA favors the formation of core-shell structures when prepared by way of feeding in bulk (droplet nucleation). By contrast, the hydrophobic monomer BA incorporated improves the encapsulation of silica particles due to the lower Tg thus lowering the resistance to penetration of the silica particles when the monomers are fed dropwise (homogeneous nucleation). The results derived from TEM and SEM technologies show that the morphologies of the as-prepared acrylate/nano-SiO_2_ composites of different feed ratio of MA/BA change from core-shell structure to bare acrylic spheres as the increase of feed ratio of MMA/BA. It can be concluded that the oil-water interfacial tension, contact angle and the viscosity of disperse phase provide criteria for the formation of core-shell structure. The composite emulsion with well core-shell-structured particles is stable. The control of the organization of silica particles at the interface can modify emulsion end-use properties.

## Competing interests

The authors declare that they have no competing interests.

## Authors’ contributions

JJ and SS carried out the preparation and main characterization of different samples and drafted the manuscript. FW, ZL, JL, YS, and YJ participated in the design of the study and the manuscript modification. All authors read and approved the final manuscript.
